# Two- and Three-Dimensional Spectrofluorimetric Qualitative Analysis of Selected Vegetable Oils for Biomedical Applications

**DOI:** 10.3390/molecules25235608

**Published:** 2020-11-28

**Authors:** Aleksandra Zielińska, Konrad Kubasiewicz, Krzysztof Wójcicki, Amélia M. Silva, Fernando M. Nunes, Marlena Szalata, Ryszard Słomski, Piotr Eder, Eliana B. Souto

**Affiliations:** 1 Institute of Human Genetics, Polish Academy of Sciences, Strzeszyńska 32, 60-479 Poznań, Poland; zielinska-aleksandra@wp.pl (A.Z.); ryszard.slomski@up.poznan.pl (R.S.); 2 Department of Pharmaceutical Technology, Faculty of Pharmacy, University of Coimbra, Pólo das Ciências da Saúde, Azinhaga de Santa Comba, 3000-548 Coimbra, Portugal; 3 Faculty of Chemistry, Adam Mickiewicz University in Poznań, Uniwersytetu Poznańskiego 8, 61-614 Poznań, Poland; kkubasiewicz@amu.edu.pl; 4 Institute of Quality Science, Poznań University of Economics and Business, Aleje Niepodległości 10, 61-875 Poznań, Poland; krzysztof.wojcicki@ue.poznan.pl; 5 Department of Biology and Environment, University of Trás-os-Montes e Alto Douro, UTAD, Quinta de Prados, P-5001-801 Vila Real, Portugal; amsilva@utad.pt; 6 Centre for Research and Technology of Agro-Environmental and Biological Sciences (CITAB), University of Trás-os-Montes and Alto Douro (UTAD), Quinta de Prados, P-5001-801 Vila Real, Portugal; 7 Department of Chemistry, University of Trás-os-Montes e Alto Douro, UTAD, Quinta de Prados, P-5001-801 Vila Real, Portugal; fnunes@utad.pt; 8 Chemistry Research Centre—Vila Real (CQ-VR), Food and Wine Chemistry Laboratory, University of Trás-os-Montes and Alto Douro (UTAD), Quinta de Prados, P-5001-801 Vila Real, Portugal; 9 Department of Biochemistry and Biotechnology, Poznań University of Life Sciences, Dojazd 11, 60-632 Poznań, Poland; szalata@up.poznan.pl; 10 Department of Gastroenterology, Dietetics and Internal Diseases, Poznan University of Medical Sciences, Przybyszewskiego 49, 60-355 Poznań, Poland; piotr.eder@op.pl; 11 CEB—Centre of Biological Engineering, University of Minho, Campus de Gualtar, 4710-057 Braga, Portugal

**Keywords:** vegetable oils, fatty acids, fluorescent ingredients, antioxidant components, 2D and 3D dimensional emission spectra

## Abstract

Vegetable oils obtained from different plants are known for their beneficial effects on prophylaxis and supportive treatment of a great deal of inflammatory-mediated conditions. Their wide range of saturated and unsaturated fatty acids, and the presence of other ingredients (e.g., tocopherols, chlorophylls), provide them with anti-inflammatory, antioxidant and anticancer properties, which are worth being exploited. In this study, we have carried out the spectrofluorometric analysis of selected vegetable oils, namely apricot (*Prunus armeniaca*) kernel oil; blueberry (*Vaccinium* spp.) seed oil; argan (*Argania spinosa*) nut oil; kiwi (*Actinidia deliciosa*) seed oil; grape (*Vitis vinifera*) seed oil; evening primrose (*Oenothera biennis*) oil and meadowfoam (*Limnanthes alba*) seed oil, with the purpose to detect their fluorescent ingredients for further identification and bioactivity comparison. The obtained two- (2D) and three-dimensional (3D) emission spectra offered a complete description of the fluorescent components of the mixture and revealed different features for studied oils.

## 1. Introduction

There is a growing body of evidence on the potential biomedical uses of different vegetable oils [1]. In this study, apricot (*Prunus armeniaca*) kernel oil; blueberry (*Vaccinium* spp.) seed oil; argan (*Argania spinosa*) nut oil; kiwi (*Actinidia deliciosa*) seed oil; grape (*Vitis vinifera*) seed oil; evening primrose (*Oenothera biennis*) oil; meadowfoam (*Limnanthes alba*) seed oil were chosen for spectrofluorimetric qualitative analysis in order to detect the active ingredients that determine their biomedical properties and further clinical use.

*Prunus armeniaca* L. (apricot) is a tree belonging to the *Rosaceae* family. An apricot kernel is a source of multiple proteins, fiber and cyanogenic compounds [2]. It contains high amounts of oleic (65 wt.%) and linolenic acids (30 wt.%), which are the main components of the oil fraction [3]. Many attempts have been made to use the apricot kernel oil in clinical practice, taking into account its antioxidant (radical scavenging) and antimicrobial properties. Recent data have shown that it can have a beneficial effect on different inflammatory-mediated conditions, such as reduction of the inflammatory activity of ulcerative colitis (UC) [2], a chronic and incurable condition of unknown etiology. The most characteristic pathophysiologic phenomenon in ulcerative colitis is the inflammatory infiltration in the superficial layers (mainly in the mucous membrane) of the colon [4]. In a rat model of chemically induced (trinitrobenzene sulfonic acid) ulcerative colitis, Minaiyan et al. (2014) [2] have used apricot extract and extract/oil and compared it with a standard treatment (prednisolone). The authors reported that both, on the macroscopic and microscopic levels, showed a significant improvement in disease activity. Amygdalin present in apricot kernel oil was shown to suppress the transcriptional mRNA, encoding different proinflammatory mediators like tumor necrosis factor-alpha (TNF-α) and interleukin-1beta (IL-1β). The study results translate the need for further assessment of the clinical utility of apricot extracts and oil in different inflammatory-mediated conditions like UC [2,4].

Blueberry seed oil, rich in alpha-linolenic acid, carotenoids, zeaxanthin, tocopherols, lutein, and cryptoxanthin, showed antioxidant activity in vitro [5], and many other biological properties which have been attributed to the oil compounds [5]. Research on the blueberry seed oil bioactivities is, however, limited to its antioxidant activity. Fruits show multipotential properties, due their high content of anthocyanins (from 25 to 495 mg/100 g of blueberries) and other polyphenols (48 mg up to 304 mg/100 g of fresh fruit weight—up to 0.3 wt.%), which are mainly present on their peels and pulp [6,7].

Another oil with multiple beneficial effects on human health is the argan (*Argania spinose*) oil, especially on skin repair, due to anti-inflammatory and wound healing properties [8,9,10]. It contains high amounts of triglycerides and poly-unsaturated fatty acids (PUFA), namely oleic and linoleic acids, and sterols (about 82 up to 104 mg/100 g oil), triterpene alcohols (lupane, ursane and oleanane derivatives), tocopherols and polyphenols [8,11]. It was also shown that virgin argan oil, rich in phenolic compounds, fatty acids, tocopherol, has the potential to protect from injuries caused by reactive oxygen species (ROS), as it inhibits LDL-oxidation, and could be used to prevent cardiovascular disease [12]. Since ROS are important pathologic factors in different inflammatory conditions, it seems that argan oil could have supportive effects, especially on diabetic patients and in obesity [13,14,15].

Kiwifruit (*Actinidia chinensis* Planch) seed oil showed beneficial influence on different aspects of metabolic and inflammatory-mediated phenomena, in a mice model of obesity induced with high-fat diet [16]. Qu et al. (2019) [16] showed that a high-fat diet combined with kiwifruit seed oil (1.0–3.0 mL/kg·bw) for 12 weeks reduced bodyweight by decreasing the amount of fatty tissue and serum lipid concentration, improving insulin resistance and decreasing glycaemia. Molecular analysis revealed a significant decrease in mRNA levels of multiple proinflammatory mediators, like TNF-α, interleukine-6 (IL-6), or cyclooxygenase-2 (COX-2) and inducible nitric oxide synthase (iNOS), and down-regulation of several genes expression involved in thermogenesis, as peroxisome proliferator-activated receptor gamma (*PPAR-*γ), peroxisome proliferator-activated receptor γ coactivator-1 (*PGC-1*) [16]. Additionally, kiwifruit seed oil significantly ameliorated gastrointestinal microbiota by decreased the *Firmicutes*-to-*Bacteroidetes* ratio, revealing the potential to change the typical dysbiotic profile of gut microbiota in obesity [16].

Grape (*Vitis vinifera* L.) seed oil was shown to exhibit crucial properties, including the improvement of oxidative stress parameters and several metabolic disturbances [8,17]. In a study with overweight and obese patients, randomly selected to consume 15% of energy from grape seed oil or sunflower oil together with a weight loss diet for 8 weeks, grape seed oil consumption decreased TNF-alpha levels, improved insulin resistance and decreased high sensitive C-reactive protein concentration, being the decrease of this latter more pronounced when compared to the sunflower oil effect [17]. Grape seed oil also improves wound healing and presents antibacterial and antioxidant activity [8].

Evening primrose (*Oenothera biennis* L.) oil is obtained from the seeds and is rich in essential fatty acids (γ-linolenic acid and linoleic acid), tocopherols and sterols [18,19]. Montserrat-de la Paz et al. (2012) have reported that evening primrose oil induces anti-inflammatory effects [19]. It can be also used in arthritic and rheumatic conditions, skin disorders (e.g., atopic dermatitis, psoriasis), diabetic neuropathy and other conditions [18]. Due to its analgesic properties, it can help in reducing menstrual pain, and abdominal pain in the course of irritable bowel syndrome [20]. Majdinasab et al. (2018) [21] suggested that evening primrose oil could be considered as adjuvant therapy in multiple sclerosis only alongside the standard treatment [21].

The meadowfoam (*Limnanthes alba*) is grown as annual crop in Oregon (USA). Oil extraction from seeds allows seed meal to be obtained, containing glucolimnanthin—a glucosinolate that can be then converted into 3-methoxybenzyl isothiocyanate (ITC) and 3-methoxyphenylacetonitrile (nitrile) in the presence of the enzyme myrosinase, used as a biopesticide [22]. Concerning the oil composition, *Limnanthes alba* is the richest known source of Δ5-unsaturated very long chain fatty acids (VLCFAs) amounting to 87–98 wt.% [23], including mainly monounsaturated fatty acids, namely gondoic acid (C20:1, ω-9) and erucic acid (C22:1, ω-9), but also vitamins A and E, both having antioxidant properties [24,25,26]. Therefore, meadowfoam seed oil is especially used in cosmetic formulations [27]. The content in high amounts of long chain fatty acids (C20–22) ensures the oil a high lubricity and a good penetration rate onto the skin. These fatty acids help the *Limnanthes alba* oil to resist oxidation, making it a stable oil [28]. Thus, it can have beneficial effects when applied topically, namely on skin and hair [28].

Fluorescence analysis is used in qualitative and quantitative determinations, since fluorescent methods are characterized by high sensitivity, accuracy and selectivity. The selectivity of the method results from the fact that not all compounds fluoresce and from the possibility of determining the appropriate wavelength of excitation and emitted radiation. Fluorescence emitted by vegetable oils is the result of the presence of compounds from the group of chlorophylls, phenols and tocopherols and other fluorescent components, which are excited at specific wavelengths [29,30]. The concentration of the oil samples, and the profile of molecules, has a pronounced effect on the fluorescence spectra [31], for example in tocopherol quantification, higher oil concentrations correspond to stronger tocopherol emission [31].

Although the analyzed oils are not commonly treated as luminophores, they exhibit fluorescence and therefore, this phenomenon was investigated. Particularly, the first spectrofluorometric analysis of meadowfoam seed oil was performed, thanks to which the antioxidant and antiaging properties of the oil have been confirmed [32]. In our study, fluorescence spectroscopy followed by multivariate treatment of the spectral data was used to establish the composition of selected vegetable oils, as reported by Wójcicki et al. (2013, 2015) [33,34]. This study aimed to develop a method of preparing oils for spectroscopic studies and evaluate the feasibility of total fluorescence spectroscopy for fingerprinting, identification and quality monitoring of oils.

## 2. Results and Discussion

**Figure 1:**
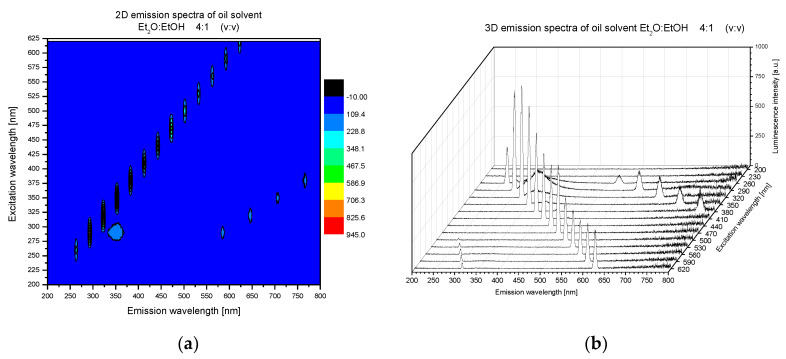
Correlative spectra of solvent (diethyl ether/ethanol, 4/1, *v*/*v*) as a blank probe: in two- (2D) (**a**) and three-dimensional (3D) (**b**) presentation.

Figure 1 shows the correlative spectra of solvent (diethyl ether/ethanol, 4/1, *v*/*v*) used as blank. The visible peaks displayed as a cross-line are a common feature from not using any filters and also from the lamp. The peaks are due to Raman scattering from the solvent molecules and second order diffraction.

In these spectra, an additional cross-line of peaks is visible (right-hand side of three-dimensional (3D) emission spectra (Figure 1), which is linked to the second harmonic generation in terms of high lamp energy, wide shutters and no other interferences.

It is worth to underline that a moderate emission band at 350 nm was observed in the solvent spectrum under 290 nm excitation. The fluorescence of both ether and ethanol are known, but they occur in other ranges [35,36]. Thus, the forming of a new ether–ethanol complex is postulated in this system, which is very likely to occur due to cluster-forming properties of these two compounds [36,37].

The band in the range of 325–375 nm under excitation with 275–300 nm confirmed the formation of the new complex between molecules of diethyl ether and ethanol, which is in agreement with literature and also with distribution of an electric charge in both molecules [36,37]. Additionally, ethanol-diethyl ether is also used as blended fuel or solvent [38].

Using spectrofluorometric analysis, two-dimensional (2D) and 3D emission spectra of studied oil have been obtained (Table 1). In fact, these are the same correlative spectra in 2D (color) and 3D (peaks), showing complementary information. Since the total fluorescence spectra of the oils show the characteristic bands of vegetable oils, the short-wavelength band in the excitation range of 270–310 nm and the emission range of 300–360 nm, which is presented in most samples of oils (A, B, C, G), could be attributed to the tocopherol emission, as reported in the literature [39]. From the recorded intensity, it could be stated that tocopherol is one of the main ingredients. It also allows us to distinguish ingredients and other oils, as tocopherols are commonly used as antioxidant additives in pharmaceutical and food industries. Table 1 shows the obtained results.

The long wavelength band observed in the excitation range of 330–450 nm and emission between 660 to 700 nm could be ascribed as the emission of chlorophyll and pheophytins [30]. That band strongly appeared in sample F (evening primrose oil) due to the fact that, among the studied oils, it is one that contains this ingredient (Table 2). In the spectra of oil E, it was also interesting to see a very weak band that could be also interpreted as very diluted amount of some chlorophylls.

The significant differences between the oil spectra are also observed in the intermediate spectral zone (emission range 370–460 nm and excitation at 300–350 nm). Only a low or very low intensity emission band appears in this region in the spectrum of samples D (kiwi oil), E (grape seed oil) and F (evening primrose oil). The chemical compounds associated with the emission observed in oils in this range have not been unambiguously identified so far. However, in several studies it was suggested that this emission belongs to oxidation products [40].

The three-dimensional spectra, known as the excitation-emission matrix or total luminescence spectra, as well as correlative spectra (in which one axis represents the excitation wavelength, the second the emission wavelength and the third the intensity), were measured for oil samples. These spectra give a complete description of the fluorescent components of the mixture and exhibit different features for the studied oils (Table 2).

In order to identify tocopherols, the corresponding pure standard compounds have been added to the samples and the oils should have been reanalyzed for comparison. However, from the spectral profile, it can be speculated that, tocopherols occurred in the samples of *Prunus armeniaca* (apricot) kernel oil [41], blueberry oil [42], *Argania spinosa* (argan) nut oil [43] as well as *Limnanthes alba* (meadowfoam) seed oil [44], while chlorophylls and pheophytins occurred only for *Oenothera biennis* (evening primrose) oil [45]. Furthermore, oxidation products were determined for kiwi seed oil (D), grape seed oil (E) and evening primrose oil (F). It can thus be assumed that D, E and F oils have been thermally treated (refined). This is evidenced by the lack of intensive bands typical of cold pressed oils, while the observed bands have been assigned to the oxidation products (Table 1). These oils (see Table 3 for specifications) should emit some characteristic bands of their specific compounds. The absence of these bands and the presence of the oxidation bands in the intermediate spectral zone (emission range 370–460 nm and excitation at 300–350 nm) demonstrate that a thermal treatment has been performed.

## 3. Methodology

### 3.1. Materials

Vegetable oils, namely: apricot (*Prunus armeniaca*) kernel oil; blueberry(*Vaccinium* spp.) seed oil; argan (*Argania spinosa*) nut oil; kiwi (*Actinidia deliciosa*) seed oil; grape (*Vitis vinifera*) seed oil; evening primrose (*Oenothera biennis*) oil; meadowfoam (*Limnanthes alba*) seed oil were sourced from The Kerfoot Group Ltd. (Northallerton, UK), while cold-pressed *Limnanthes alba* (meadowfoam) seed oil was purchased from Natural Plant Products, Inc. (Salem, OR, USA). The composition of studied oils is summarized in Table 3.

### 3.2. Fluorescence Measurements

Due to high absorption, the investigated oils were previously diluted in order to obtain a transparent dispersion. The water was excluded. Several organic solvents were selected, namely, pure ethanol, acetone, diethyl ether, cyclohexane, and cyclohexanone. Each of these reagents caused nontransparent emulsions/suspensions. Hence, regarding polarity, their mixtures in various ratio were tested. Finally, the solving phase was a mixture of diethyl ether (Et_2_O) and ethyl alcohol (EtOH) in volume ratio 4:1. As a result, 5% (*w*/*v*) of oils solutions were prepared in 10 mL flasks.

After preparation of the oil solutions, the correlative spectroscopy was performed. In the first step, for each oil the emission spectra were registered in the range of 200–800 nm (0.2 nm resolution) under excitation wavelengths in the range of 200–620 nm (with 30 nm steps) using traditional Fluorescence Spectrophotometer F-7000 (Hitachi High-Tech, Shimadzu, Kyoto, Japan)), equipped with long-life 150 W Xe lamp, monochromator diffraction grating (900 lines/mm), measured in the range of 200–800 nm with a resolution of 1 nm, and a detection system: photomultiplier. Registered correlative spectra of pure solvent and spectra of investigated oils were presented as 2D and 3D charts.

## 4. Conclusions

In this study, a method of preparing oils for spectroscopic studies using ethanol and diethyl ether was described. Selected vegetable oils: apricot (*Prunus armeniaca*) kernel oil; blueberry (*Vaccinium* spp.) seed oil; argan (*Argania spinosa*) nut oil; kiwi (*Actinidia deliciosa*) seed oil; grape (*Vitis vinifera*) seed oil; evening primrose (*Oenothera biennis*) oil; meadowfoam (*Limnanthes alba*) seed oil were characterized using two- (2D) and three-dimensional (3D) spectrofluorometric analysis. The 2D and 3D emission spectra were collected for the wavelengths, showing the highest intensities. Broad-spectrum characteristics were obtained by collecting 2D and 3D correlation spectra for each oil and for the solvent phase. The quality of the spectra can be improved by limiting the measuring range. As the available filters of spectrofluorometer are responsible for a strong luminescence on their own and under the used excitation, no filters were applied for the analysis of the oils. The spectrum appears at x = y bands, caused by the Raighley scattering effect. Solvent phase bands were observed in each spectrum of the oil solution, thus indicating a metastable complex formed in the solvent, together with a charge transfer effect. Based on the obtained results, a total fluorescence spectroscopy can be used for qualitative characteristic of the respective oils.

## Figures and Tables

**Table 1:** 2D (left) and 3D (right) emission spectra of studied oils: (A) apricot (*Prunus armeniaca*) kernel oil; (B) blueberry oil; (C) argan (*Argania spinosa*) nut oil; (D) kiwi seed oil, (E) grape seed oil; (F) evening primrose (*Oenothera biennis*) oil; (G) *Limnanthes alba* (meadowfoam) seed oil.

^Sample^	2D	3D
A	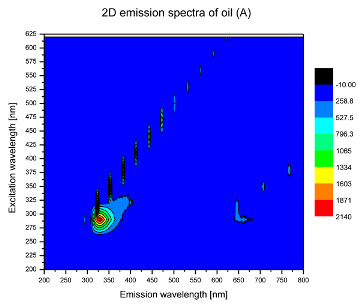	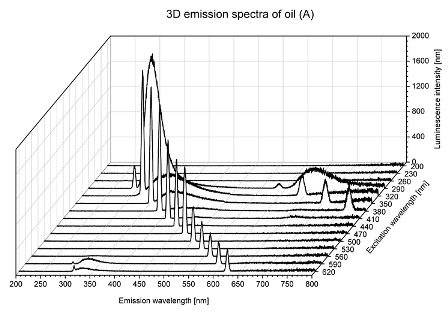
B	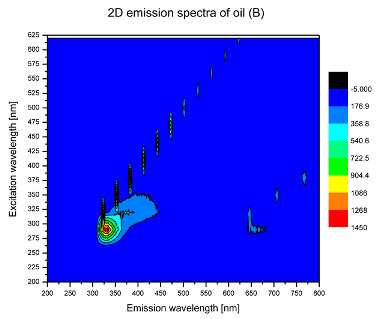	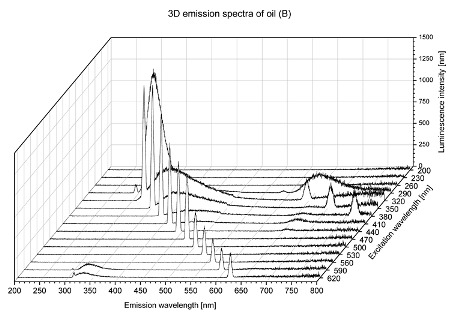
C	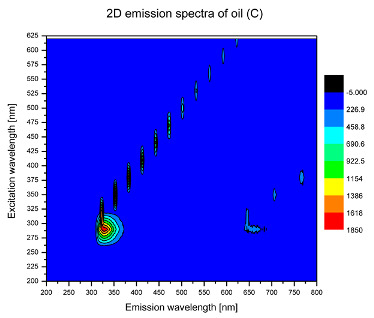	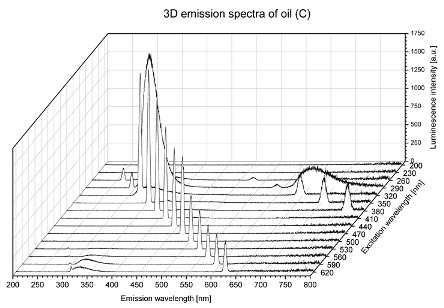
D	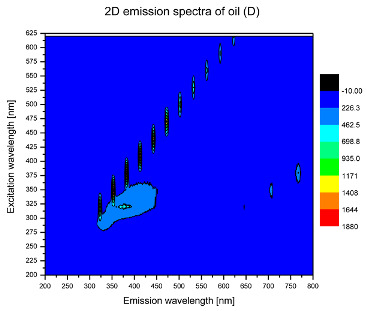	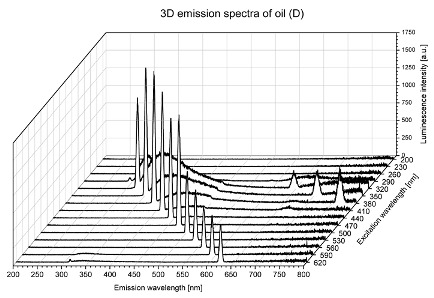
E	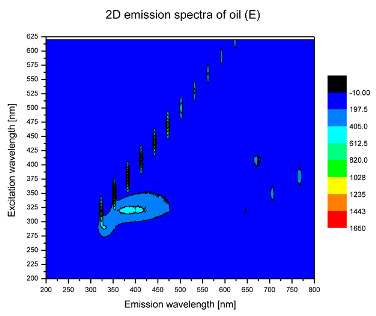	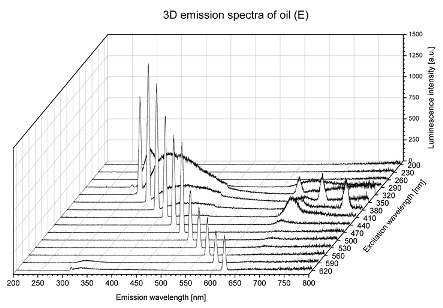
F	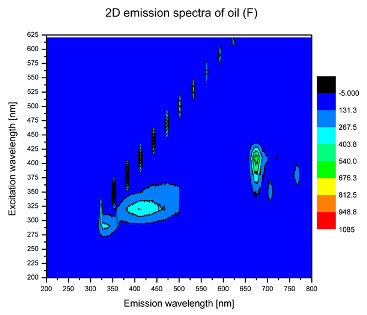	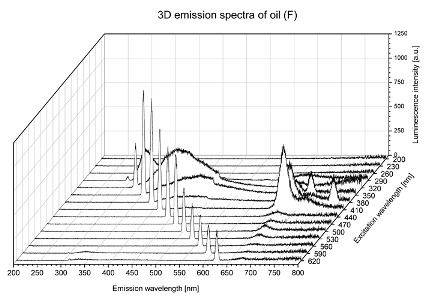
G	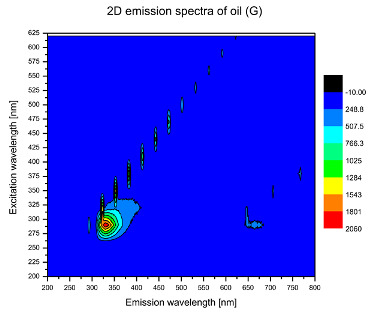	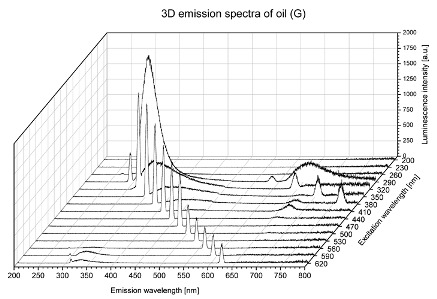

**Table 2:** Spectral profile of studied oils.

Oil	A	B	C	D	E	F	G
Plant	Apricot	Blueberry	Argan	Kiwi	Grape	Primrose	Meadowfoam
**tocopherols**	**x**	**x**	**x**		**x**	**x**	**x**
(excitation range of 270–310 nm and the emission range of 300–360 nm)							
**chlorophylls and pheophytins**						**x**	
(excitation range of 330–450 nm and emission range between 660 to 700 nm)							
**oxidation products**				**x**	**x**	**x**	
(excitation range at 300–350 nm and emission range 370–460 nm)							

**Table 3:** The composition profile of studied oils (based on the references: [1,2,3,4,5,6,7,8,9,10,11,12,13,14,15,16,17,18,19,20,21,22,23,24,25,26,27,28]).

Sample	Oil Name	Fatty Acids and Other Ingredients	Health Benefits Briefly
**A**	apricot (*Prunus armeniaca*) kernel oil	PA (5); LA (30); OA (65); tocopherols; phytosterols	improving balance of destructive cytokines and reduction of toxic stress in the bowel cells; antioxidant and antimicrobial activities
**B**	blueberry (*Vaccinium* spp.) oil	PA (5.7); SA (2.8); ALA (25.1); LA (43.5); OA (22.9); anthocyanins; polyphenols; tocopherols; tocotrienols; carotenoids	improves inflammatory markers; promotes cardiovascular health; support healthy aging and gut health; radical scavenging activity
**C**	argan (*Argania spinosa*) nut oil	PA (12.8); SA (5.8); ALA (0.5); LA (33); OA (46.6); polyphenols, tocopherols; antioxidants; sterols; carotenoids; xanthophylls; squalene	cardioprotective properties; used in the treatment of skin infections; cures skin pimples, juvenile acne, and chicken pox pustules; reduces the rate of appearance of wrinkles; fights dry skin and dry hair; choleretic, hepatoprotective, useful to treat hypercholesterolemia and atherosclerosis
**D**	kiwi (*Actinidia deliciosa*) seed oil	PA; SA; ALA (67); LA (14–57); OA (12); tocopherols; tocotrienols	aids in the relief of itchy, scaly, irritated skin conditions, e.g., eczema/psoriasis; improves skin elasticity, reduces skin lines, dryness, wrinkles, enhances regeneration of skin cells
**E**	grape (*Vitis vinifera*) seed oil	PA; SA; ALA (0.5); LA (72–85); OA (10); tocopherols; tocotrienols; phenolic compounds [flavonoids, carotenoids, phenolic acids, tannins, stilbenes]; resveratrol; quercetin; procyanidins; carotenoids; phytosterols; gallic acid; catechin; epicatechin; procyanidins; proanthocyanidins	maintenances the ratio between anti and pro-inflammatory cytokines on serum (TNF-α/IL-10); reduces oxidative stress, decreases low-density lipoprotein (LDL) levels; inhibits lipid oxidation; anti-inflammatory and antioxidant capabilities; has a toxicity effect on some pathogens, suggesting an antimicrobial feature; cardioprotective and anticancer effects;
**F**	evening primrose (*Oenothera biennis*) oil	PA (6.2); SA (1.8); ALA (<2); LA (75); GLA (9–10); OA (5.4); phytosterols [4-desmethylsterols, erythrodiol and uvaol]; phenols [mainly ferulic acid]; tocopherols	widely used as a dietary supplement; helps in rheumatic and arthritic conditions, atopic dermatitis, psoriasis, premenstrual and menopausal syndrome - although there is little evidence to support these uses; inhibitory effect on leukotriene synthesis; implicates various inflammatory and immunologic pathogeneses
**G**	meadowfoam (*Limnanthes alba*) seed oil	EA (63); EU (16–24); C22:1 (17); glucolimnanthin (3–4), methoxylated benzyl glucosinolate (a phenylalanine-derived);	anti-microbial properties; its exceptional oxidative stability and lubricity; ameliorates abnormal skin conditions

Approximate percentages of individual ingredients are given in brackets. Abbreviations of saturated and unsaturated fatty acids: ALA—α-linolenic acid; EA—eicosenoic acid; EU—erucic acid; GLA—γ-linolenic acid; LA—linolenic acid; OA—oleic acid; PA—palmitic acid; SA—stearic acid.
